# Clinical Features and Treatment of Intra-Tendinous Suture Reaction Following Achilles Tendon Repair Using Nonabsorbable Suture Material: A Retrospective Case Series Study

**DOI:** 10.3390/ijerph191912897

**Published:** 2022-10-08

**Authors:** Jahyung Kim, Hwa-Jun Kang, Bom-Soo Kim, Yu-Mi Kim, Hyong-Nyun Kim, Jae-Yong Park, Young-Rak Choi, Altanzul Bat-Ulzii, Jaeho Cho

**Affiliations:** 1 Department of Orthopaedic Surgery, Armed Force Yangju Hospital, Yangju 11429, Korea; 2 Department of Orthopedic Surgery, Dongtan Sacred Heart Hospital, Hallym University College of Medicine, Hwaseong 18450, Korea; 3 Department of Orthopedic Surgery, Inha University Hospital, Incheon 22332, Korea; 4 Department of Orthopedic Surgery, Sanbon Hospital, Wonkwang University College of Medicine, Gunpo-si 15865, Korea; 5 Department of Orthopedic Surgery, Kangnam Sacred Heart Hospital, Hallym University College of Medicine, Seoul 07441, Korea; 6 Department of Orthopedic Surgery, Hallym Sacred Heart Hospital, Hallym University College of Medicine, Anyang-si 14068, Korea; 7 Department of Orthopedic Surgery, Asan Medical Center, University of Ulsan College of Medicine, Seoul 03924, Korea; 8 Institute for Skeletal Aging and Orthopedic Surgery, Chuncheon Sacred Heart Hospital, Hallym University, Chuncheon 24253, Korea; 9 Department of Orthopedic Surgery, Chuncheon Sacred Heart Hospital, Hallym University, Chuncheon 24253, Korea

**Keywords:** Achilles tendon, nonabsorbable suture material, foreign body reaction, granuloma formation, sinus formation

## Abstract

We aimed to investigate the clinical manifestations, radiological findings, and therapeutic outcome of treatment for patients with surgically confirmed foreign body reaction following an Achilles tendon repair using non-absorbable suture material. Eight consecutive patients who were confirmed as having an intra-tendinous suture foreign body reaction in the histopathological report were enrolled in this study. Medical records of all patients in terms of clinical and radiological features were retrieved. Also, the outcome of treatment was evaluated at a follow-up of at least 12 months. All the patients complained of pain and a palpable mass around a previous surgical site at mean 25.1 months (range, 4–72 months) after the initial surgery. Magnetic resonance imaging (MRI) or ultrasound were used to detect the lesion. All the patients underwent surgical excision of foreign body reaction tissue and primary repair using absorbable suture material. After the treatment, the wounds were healed completely in all, and the average FAOS (foot and ankle outcome score) was 91.32 at mean follow-up for 22.4 months. In conclusion, intra-tendinous suture reaction is a rare complication following an Achilles tendon repair using nonabsorbable suture material, but it can be treated adequately with only surgical excision of foreign body reaction tissue and primary repair using absorbable suture material.

## 1. Introduction

The primary goals in managing an acute Achilles tendon rupture are to promote permanent tendon healing at the correct length and to ensure a rapid return to normal function [1]. In order to fulfill these goals and establish superior clinical outcomes, a myriad of therapeutic approaches have been attempted and analyzed to date. Of the many issues toward an Achilles tendon treatment, however, the selection of suture material in surgical repair is known to be dependent on a surgeon’s preference [2].

In order to provide adequate fixation strength within the tendon during the critical healing period, nonabsorbable, multifilament sutures have been preferred [3,4]. Recently, in fact, multiple comparative trials have revealed that absorbable suture materials may guarantee equivalent fixation strength and postoperative outcomes with nonabsorbable materials [5,6]. Considering this, the main concern of using a nonabsorbable material is that it permanently remains within the tendon, which may eventually lead to either an infection or a foreign body reaction [7,8].

Quite a few reports have dealt with suture material-related foreign body granuloma after an Achilles tendon repair [7,8,9,10]. However, these studies only covered single cases and did not concretely describe specific findings of the disease entity. As a result, we aimed to serially investigate the clinical manifestations, radiological findings, and therapeutic outcome of treatment for patients with surgically confirmed foreign body reaction following an Achilles tendon repair using nonabsorbable suture material.

## 2. Materials and Methods

This retrospective study was approved by the medical ethics committee at our institution (Institutional Review Board number: CHUNCHEON 2019-02-008), and written informed consents for publication of this report were obtained from all included patients.

From January 2017 to December 2020, eight consecutive patients who were surgically confirmed to have a foreign body reaction around a previously repaired Achilles tendon were enrolled in this study. The medical records of all patients were reviewed, and the following details were retrieved: age, gender, time to visit after an initial surgery, clinical presentation, imaging findings, type of suture material used during initial surgery, operative finding, histopathologic finding, and clinical outcomes after the surgery.

In terms of diagnostic modalities, ultrasound or magnetic resonance imaging (MRI), was used. Ultrasound examinations were completed using a GE LOGIQ-E ultrasound machine (GE Healthcare, Milwaukee, WI, USA) at a frequency of 10 MHz and a depth of 3.5 cm. For MRI examinations, 1.5-T (Sonata, Siemens Healthineers, Erlangen, Germany) and 3-T (Discovery MR750w, GE Healthcare, Milwaukee, WI, USA) MRI scanners were used with a commercially available ankle coil. Postoperatively, the resected specimens were reviewed under hematoxylin and eosin staining.

## 3. Results

Overall, eight patients who developed a suture-related foreign body granuloma following an Achilles tendon repair were analyzed. Five were male and three were female, and the mean age was 46.6 ± 13.1 years (range, 24–62 years). One patient had diabetes mellitus, one patient had gout and no other significant medical comorbidities were present. One patient was a smoker. The mechanism of injury was sports-related in all except one patient from laceration.

**Figure 1:**
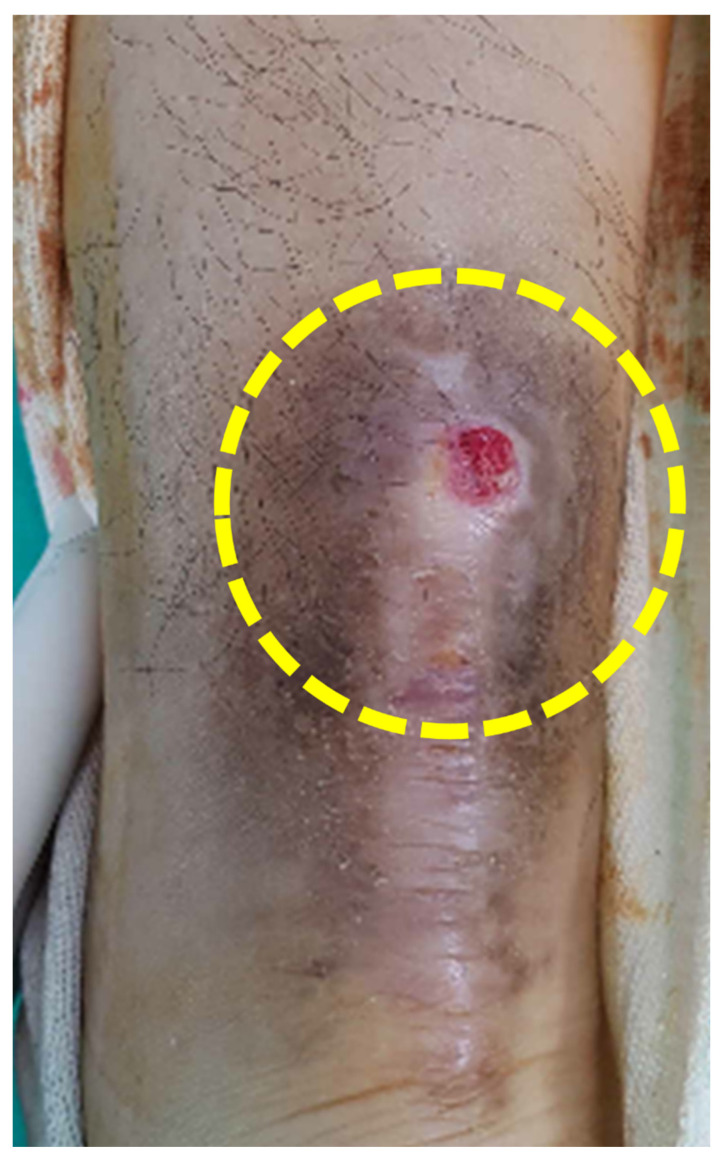
A clinical photo showing a palpable mass with mild redness (dotted circle) around a previous surgical incision.

Patients complained of pain and a palpable mass around a previous surgical site, along with mild redness and heating sensation (Figure 1). Six patients experienced wound problems such as recurrent discharge.

The average time patients visited, complaining of these symptoms, was 25.1 ± 26.4 months (range, 4–72 months) after the initial surgery. The suture material used during the surgery was nonabsorbable material (Ethibond^®^, ETHICON, Johnson & Johnson, Seoul, Korea) in all patients. Laboratory evaluation indicated normal findings in all patients.

**Figure 2:**
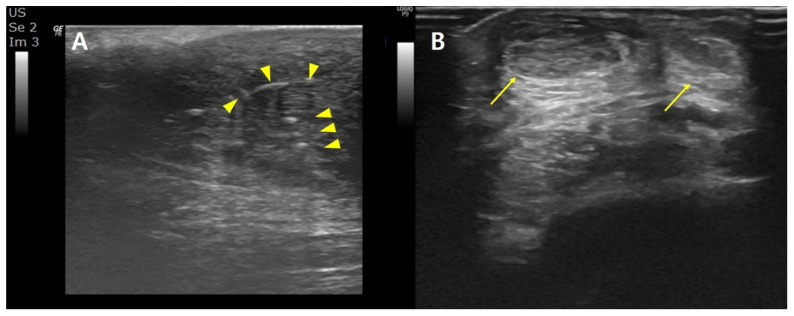
(**A**) Longitudinal and (**B**) transverse ultrasound images. Hypoechoic, heterogenous reactive cyst-like lesion around hyperechoic foci (arrow heads), indicating suture materials. Edematous and abnormally thickened tendon (arrows).

Common ultrasound characteristics included a hypoechoic, heterogenous reactive cyst-like lesion around hyperechoic foci, indicating suture materials. The tendons were edematous and abnormally thickened (Figure 2).

**Figure 3:**
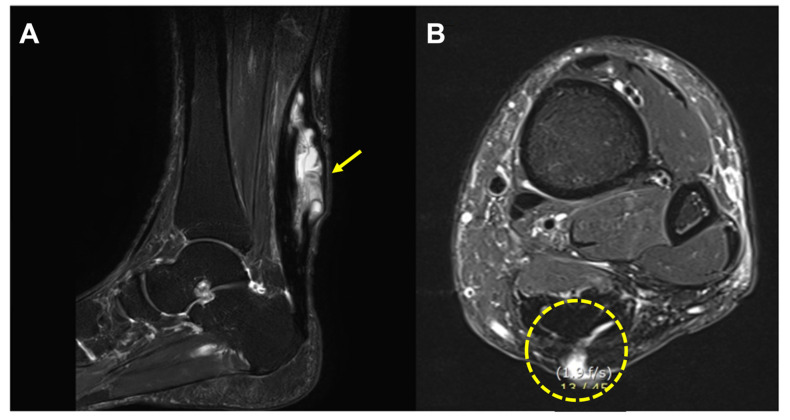
Magnetic resonance imaging (MRI) images. (**A**) A high-signal irregularly walled cystic structure (arrow) within the thickened Achilles tendon substance (patient 7). (**B**) The lesion communicated with subcutaneous space and overlying skin through draining sinuses (dotted circle).

A typical MRI finding was a high-signal, irregularly walled cystic structure within the thickened Achilles tendon substance, containing punctuate low signal foci. Also, the cystic structure was communicated with subcutaneous space or overlying skin through draining sinuses (Figure 3).

In intra-operative findings, fibrous granulomatous tissue thought to have been developed in the wake of foreign body reaction to suture materials within the repaired tendon was detected in all patients. In six patients, draining sinuses were found, which communicated from granulomatous tissue to overlying skin or subcutaneous space. No finding indicating infection was noted in all included patients. In all cases, abnormal tissue within the tendon, along with suture materials or communicating sinus tracts, was meticulously removed. Also, no additional procedure beyond tubular repair using absorbable suture material was needed to cover the defect after excision, because tendon continuity and muscle power were preserved.

**Figure 4:**
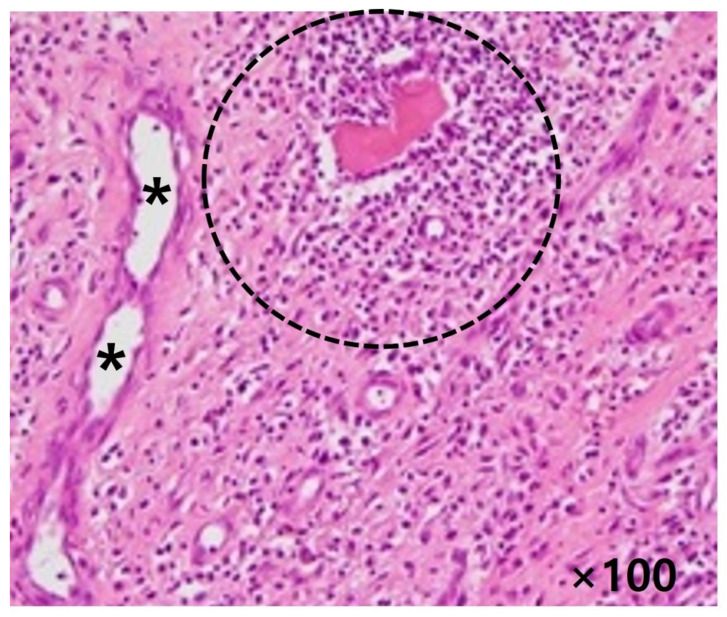
Histopathologic features of the obtained specimen. Giant cell as well as acute and chronic inflammatory cells (dotted circle) surrounding the suture materials (asterisks), indicating granulation tissue and foreign body reaction within Achilles tendon (Hematoxylin-eosin stained; magnification, ×100).

Tissue culture did not reveal any microorganism in all cases. Histopathologic examination indicated typical findings of the foreign body reaction including hemosiderine loaded macrophages, giant cells and eosinophilic infiltration, and acute and chronic inflammatory cells surrounding the suture materials. There was no evidence of infection or malignancy (Figure 4).

All patients had a fully functional tendon, and were able to resume their normal activities, without any complaints of recurrence or wound problem (Table 1).

An illustrative case of one of the patients is presented below, which summarizes the clinical course and findings of the patients in our study.

### Illustrative Case (Patient 1)

A 53-year-old male presented to our outpatient clinic, complaining of pain and a gradually enlarging mass-like lesion on the right ankle joint for the past two weeks. Two years ago, he sustained an Achilles tendon rupture after a sports activity trauma and was operated the next day in a nearby orthopedic clinic using nonabsorbable sutures (Ethibond^®^, ETHICON, Johnson & Johnson, Seoul, Korea). Since then, he suffered from intermittent wound problems around an operative scar, which were treated conservatively with dry dressing. He had no previous medical or allergic history.

Physical examination showed two solid, semimobile soft tissue masses with mild redness and localized heating sensation overlying the former operative scar. The infection parameters (white blood cell count, erythrocyte sedimentation, and C-reactive protein) were all within normal limits. The muscle power of an Achilles tendon was preserved.

**Figure 5:**
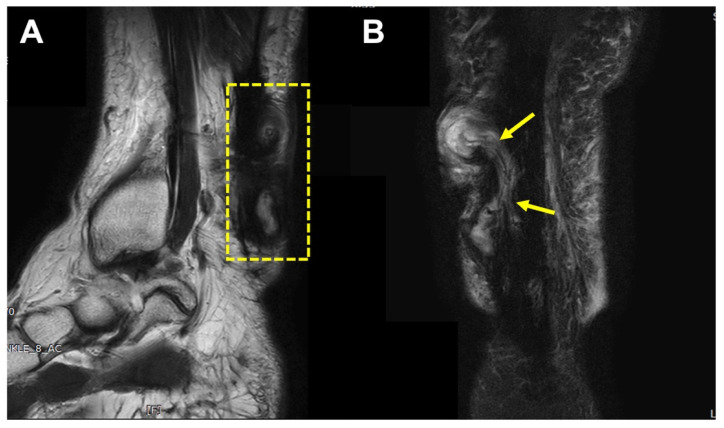
MRI findings of patient No. 1. (**A**) Two high-signaled, irregularly walled cystic structures (2 × 3, 2 × 2.5 cm sized) within the Achilles tendon substance (dotted box). (**B**) The cystic structures are communicated laterally with subcutaneous space through draining sinuses (arrows).

The patient underwent magnetic resonance imaging (MRI), which showed two high-signaled, irregularly walled cystic structures (2 × 3, 2 × 2.5 cm sized) within the Achilles tendon substance, and these structures contained multiple punctuate low-signal foci. The cystic structures were communicated laterally with subcutaneous space through draining sinuses (Figure 5). Based on these findings, our initial diagnosis was a foreign body reaction, and an operation was undertaken.

**Figure 6:**
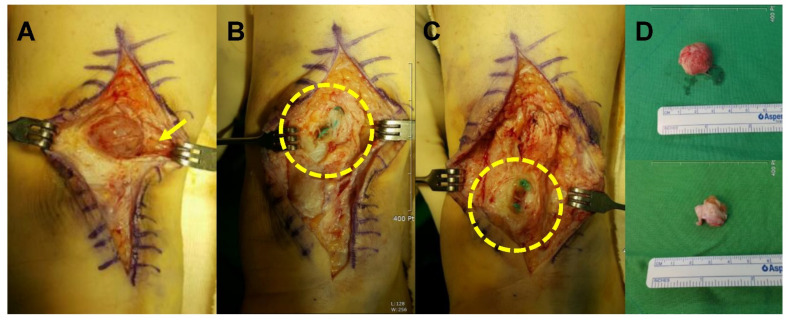
Intraoperative findings. (**A**) Two granulomas within the tendon, communicating with subcutaneous space through draining sinuses (arrow). (**B**,**C**) Granulomas filled with fibrous granulation tissue encysting the Ethibond^®^ sutures (dotted circles). (**D**) Gross images of extracted granulomas.

Following a skin incision over the previous one, the sinus tract extending from the tendon, directly down to the subcutaneous layer, could be detected. After splitting the tendon, two granulomas, filled with fibrous granulation tissue encysting the Ethibond^®^ sutures, were identified (Figure 6).

**Figure 7:**
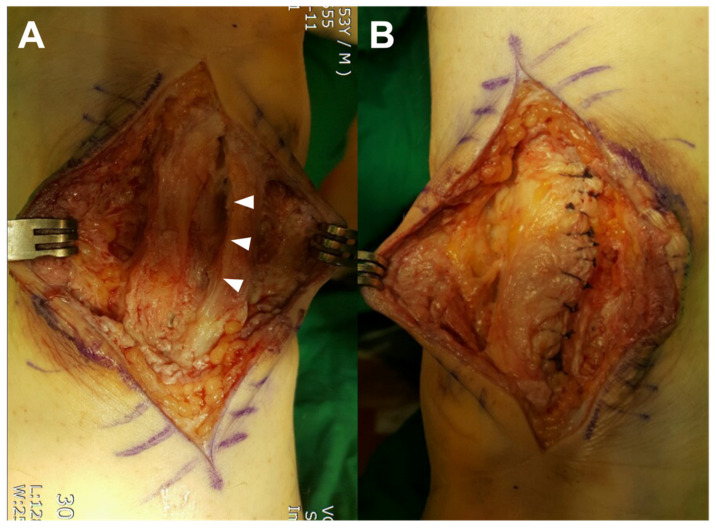
Intraoperative images describing tubular repair. (**A**) Defect made on the tendon after an excision (arrow heads). (**B**) Defect restored with tubular repair using absorbable sutures.

No evidence indicating an infection could be found. The abnormal tissue, fistula, and suture materials were meticulously excised. Because the tendon continuity and muscle strength were well preserved after the excision of suture reaction tissue including suture material, the defect on the tendon was restored with tubular repair using absorbable sutures (Figure 7).

Typical findings of foreign body reaction could be detected on histopathologic examination, and no microorganism was cultivated on tissue culture. The patient reported that the symptom subsided as early as three weeks postoperatively. At the 36-month follow-up, the patient showed satisfactory outcomes with a Foot and Ankle Outcome Score (FAOS) of 89.49, without any evidence of motor deficit, wound problem, or recurrence [11].

## 4. Discussion

Suture-related foreign body granuloma is a rare complication after an Achilles tendon repair using nonabsorbable suture material. Although less significant than re-rupture or infection, it may result in negative clinical outcomes due to pain, discomfort, or a wound problem. This study demonstrated the typical clinical course of suture material-derived foreign body granuloma, along with imaging and operative findings.

Every suture material has a potential to elicit some degree of hypersensitivity reaction within our body [12]. Factors that trigger the reaction remain uncertain, but once the immune system recognizes the suture material as a foreign body, activated macrophages attach and form giant cells to degrade it. Such a reaction may eventually create encysted sutures within the body, which may develop granulomas or draining sinuses [13].

In general, this reaction is considered a delayed-onset process and the symptoms usually appear a few months after the surgery. However, some studies dealt with patients who have symptoms much later than previously reported ones, at longest, 30 years [14,15]. Recently, Itoga et al. introduced a patient with systemic lupus erythematosus who developed the granuloma 20 years after open Achilles tendon repair [15]. In this study, four of eight patients had symptoms more than one year after their initial surgery but had no notable medical comorbidities. It is not clear which factor contributed to relatively longer onset of reaction in this study, but such delayed onset nature of reaction implies that the risk of foreign body reaction does not disappear within months unless the suture material exists within the tendon. In this context, absorbable suture materials theoretically disappear within a specific period, and may eliminate the possibility of foreign body-related symptoms. Although no direct comparison between suture materials was executed in this study, absorbable materials could be preferred in Achilles tendon repair to ensure long-term postoperative outcomes.

Of all the included patients, six cases were accompanied with draining sinuses along with the lesion, which resulted in consistent wound problems. The exact mechanism that these sinuses could be derived from suture material remains uncertain. Andrew et al. estimated that high tensile forces applied to the sutures in the Achilles tendon may result in separation of the particulate coating from the suture material, which can consequently develop a draining sinus tract [16]. Once the sinuses are detected, these should be removed along with causative suture materials within the repaired tendon.

After excision of abnormal tissues, inevitable defect within the tendon had to be covered. In order to restore the defect, Ollivere et al. considered additional flexor hallucis longus transfer to supplement the tendon [8]. In this study, however, tendon continuity was preserved intraoperatively in all cases, and no evidence of motor deficit was detected after an excision. The defects were all covered with simple tubular repair and satisfactory outcomes could be obtained. Because the foreign body granuloma usually occurs after the critical tendon healing period and does not seem to affect the strength of the primary repair, we suggest that defects could simply be repaired in a tubular manner, without additional augmentation process.

This study is limited because it is a retrospective case series with a relatively small sample size of eight patients. In addition, merely a single type of nonabsorbable suture turned out to be a causative foreign material without comparative suture types. Besides, the incidence of Ethibond^®^ suture reaction as a fatal complication following acute Achilles tendon repair could not be defined, since it was not a result of surgery performed at a single institution. Although intra-tendinous suture-related foreign body reaction is quite uncommon after Achilles tendon repair, a succeeding randomized prospective comparative trial with a larger number of patients would help to clearly validify the results of this study. Furthermore, the fact that absorbable suture-related fibromatous granuloma was not revealed in this study makes it difficult to strongly suggest that an absorbable suture material is less prone to foreign body reaction or wound complication.

## 5. Conclusions

Intra-tendinous suture-related foreign body reaction is a rare complication following an Achilles tendon repair using nonabsorbable suture material, but can result in disabling clinical outcomes. Once suspected, imaging evaluation to detect the lesion can be helpful and it can be treated adequately with only surgical excision of foreign body reaction tissue and primary repair using absorbable suture material.

## Figures and Tables

**Table 1:** Descriptive data of 8 patients.

No.	Age (yr)	Sex	Medical Conditions	Injury Mechanism	Time to Visit after Initial Surgery	Suture Material	Draining Sinus	Wound Healing Time (Weeks)	Last Follow-Up	FAOS	Recurrence
1	53	M	Gout	sport	24 mos	Nonabs	Y	4	36 mos	89.49	No
2	47	M	Diabetes	sport	9 mos	Nonabs	Y	3	12 mos	82.53	No
3	60	M	none	sport	72 mos	Nonabs	Y	2	18 mos	86.00	No
4	33	M	none	laceration	7 mos	Nonabs	Y	2	14 mos	97.19	No
5	24	F	none	sport	5 mos	Nonabs	Y	3	12 mos	97.85	No
6	52	F	none	sport	4 mos	Nonabs	Y	2	63 mos	91.04	No
7	62	M	Smoking	sport	60 mos	Nonabs	N	2	12 mos	96.05	No
8	42	F	none s	sport	20 mos	Nonabs	N	4	12 mos	90.48	No
Mean	46.6				25.1 mos			2.8	22.4 mos	91.32	

## Data Availability

The datasets used and/or analyzed during the current study are available from the corresponding author on reasonable request.
